# Structural Basis of Glycan Recognition of Rotavirus

**DOI:** 10.3389/fmolb.2021.658029

**Published:** 2021-07-08

**Authors:** Xiaoman Sun, Dandi Li, Zhaojun Duan

**Affiliations:** ^1^ National Health Commission Key Laboratory for Medical Virology and Viral Diseases, Beijing, China; ^2^ National Institute for Viral Disease Control and Prevention, China CDC, Beijing, China

**Keywords:** rotavirus, VP8* structure, glycan binding specificity, sialic acid, histo-blood group antigens, mucin cores

## Abstract

Rotavirus (RV) is an important pathogen causing acute gastroenteritis in young humans and animals. Attachment to the host receptor is a crucial step for the virus infection. The recent advances in illustrating the interactions between RV and glycans promoted our understanding of the host range and epidemiology of RVs. VP8*, the distal region of the RV outer capsid spike protein VP4, played a critical role in the glycan recognition. Group A RVs were classified into different P genotypes based on the VP4 sequences and recognized glycans in a P genotype-dependent manner. Glycans including sialic acid, gangliosides, histo-blood group antigens (HBGAs), and mucin cores have been reported to interact with RV VP8*s. The glycan binding specificities of VP8*s of different RV genotypes have been studied. Here, we mainly discussed the structural basis for the interactions between RV VP8*s and glycans, which provided molecular insights into the receptor recognition and host tropism, offering new clues to the design of RV vaccine and anti-viral agents.

## Introduction

**FIGURE 1:**
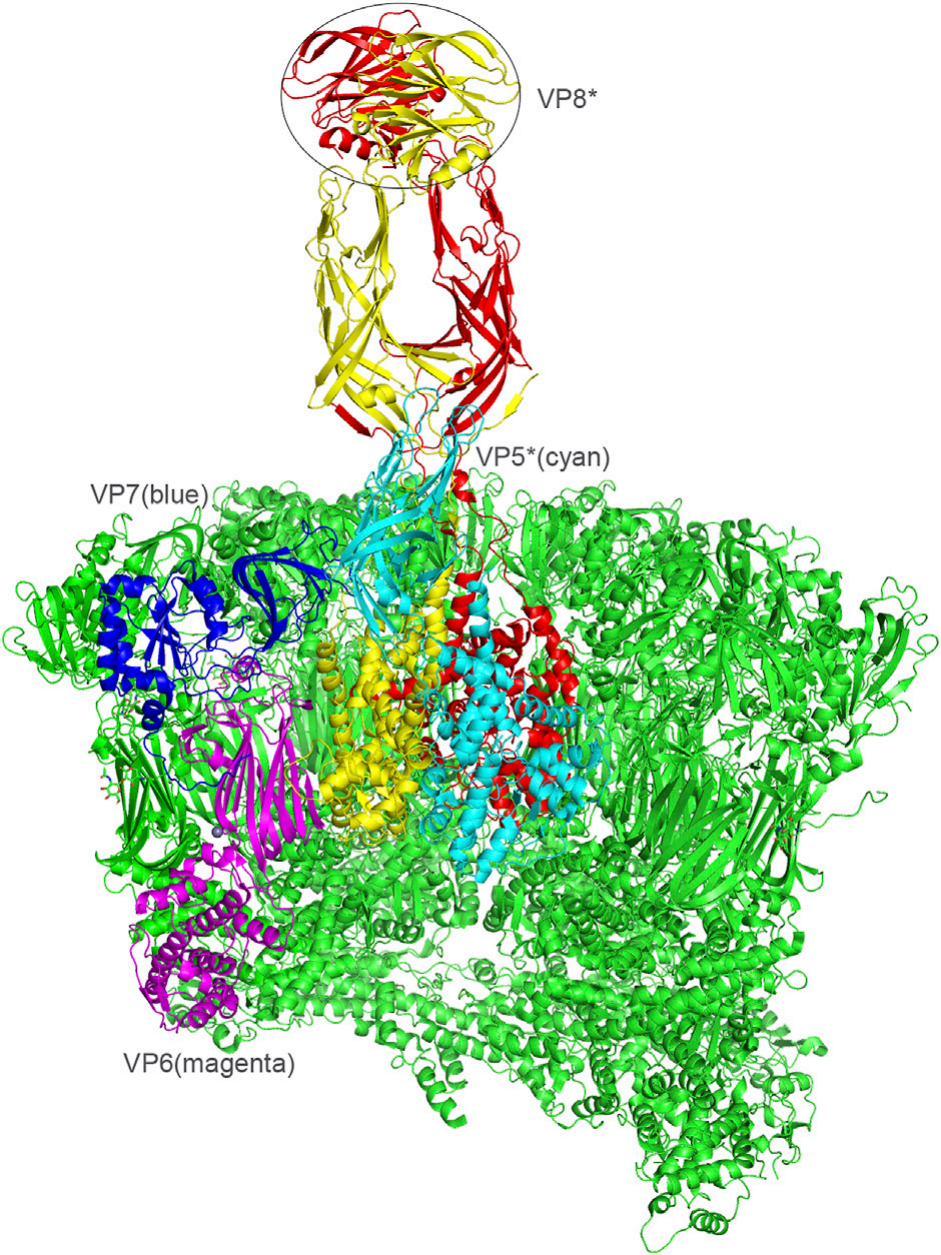
Presentation of the RV viral proteins of the middle and outer layers based on the rotavirus particle structure (PDB: 4V7Q). VP6, magenta. VP7, blue. VP4, red/yellow. VP5, cyan. VP8*, the distal domain of VP4 was shown in a circle.

Rotavirus (RV), belonging to the *Reoviridae*, is an important pathogen leading to acute gastroenteritis (AGE) in children under 5 years old and caused ∼200,000 deaths worldwide each year (Tate et al., 2016; Bányai et al., 2018). RV genome contained 11 segments of double-stranded RNA, encoding 6 structural proteins (VP) and 6 non-structural proteins (Estes and Greenberg, 2013). The RV capsid has three layers consisting of a core layer formed by VP2, an intermediate layer formed by VP6, and an outer layer formed by VP4 and VP7 (Figure 1). Based on the antigenic and molecular characteristics of VP6, RVs are currently classified into nine groups/species (A-I) and a further tentative group J (Banyai et al., 2017). Groups A, B, C, and H RVs have been identified in human infections, while other groups only cause diseases in animal species (Matthijnssens et al., 2012; Banyai et al., 2017). Among these, group A RVs (RVAs) are the most widely prevalent in humans and the leading cause of severe AGE worldwide.

VP7 is a glycoprotein and VP4 is protease-sensitive (Estes and Greenberg, 2013). VP4 extending from the VP7 shell formed the major spike protein contributing to the viral attachment and penetration (Figure 1) (Dormitzer et al., 2002b). RV was classified into G and P genotypes based on VP7 and VP4, respectively, (Matthijnssens et al., 2011). To date, no less than 37 G and 51 P genotypes of RVAs have been identified (https://rega.kuleuven.be/cev/viralmetagenomics/virus-classification). Different combinations of G and P genotypes have been reported in human infections whereas G9P[8], G1P[8], G3P[8], G2P[4], G8P[8], are the widely prevalent RVAs (Lestari et al., 2020). There is a great genetic and strain diversity of RVs, contributed by point mutations, gene rearrangement, and genetic assortment between co-circulating strains. Furthermore, interspecies transmission between human and animal RVs has been reported in different genotypes (Mukherjee et al., 2011). Though two licensed RV vaccines are effective and widely used in many countries all over the world (Anh et al., 2011; Wang et al., 2013), how effective the vaccines will be as the genetic alteration of the prevalent RVs remains unknown.

**FIGURE 2:**
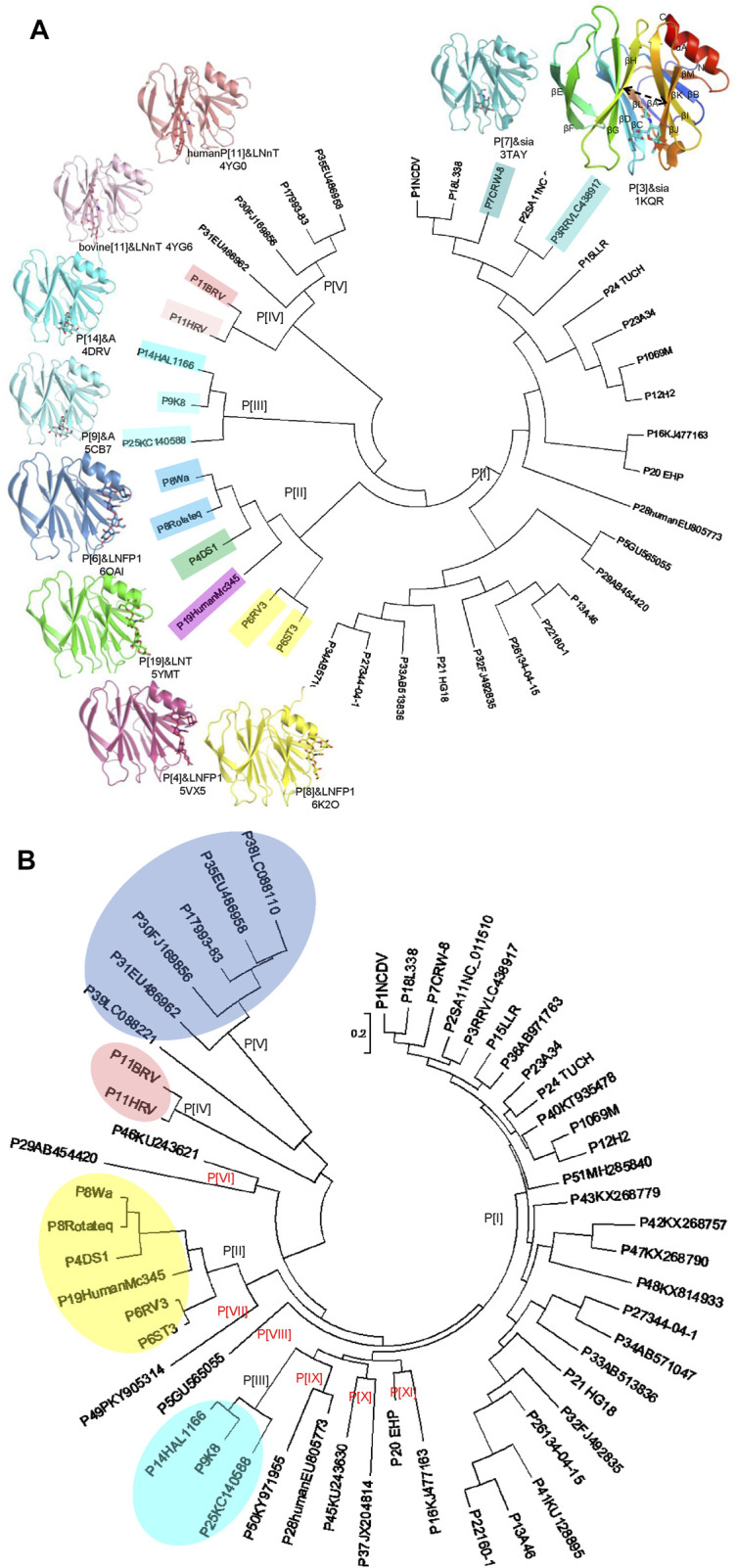
**(A)** Crystal structures of rotavirus VP8*s. Phylogenetic analysis of VP8*s of 35 RV P genotypes (circular dendrogram) was conducted by MEGA6 using the neighbor-joining method. The crystal structures of VP8*s in complex with glycans are presented. P[3], Rhesus rotavirus RRV in complex with Sia, rainbow, PDB ID: 1KQR. P[7], CRW-7 in complex with sia, teal, PDB ID: 3TAY. P[14], human rotavirus HAL1166 in complex with A-type HBGA, cyan, PDB ID: 4DRV. P[9], human rotavirus K8 in complex with A-type HBGA, Aquamarine, PDB ID: 5CB7. P[11], human neonatal rotavirus N155 in complex with type II tetrasaccharide LNnT, salmon, PDB ID: 4YG0. P[11], bovine rotavirus B223 in complex with type II tetrasaccharide LNnT, lightpink, PDB ID: 4YG6. P[6], human rotavirus RV3 in complex with LNFP1, skyblue, PDB ID: 6OAI. P[19], human rotavirus MC345 in complex with LNT, green, PDB ID: 5YMT. P[4], human rotavirus DS-1 in complex with LNFP1, warmpink, PDB ID: 5VX5. P[8], human rotavirus Rotateq in complex with LNFP1, yellow, PDB ID: 6K2O. **(B)** Phylogenetic analysis of VP8*s of 50 RV P genotypes (circular dendrogram) was conducted by MEGA6 using the neighbor-joining method. The GeneBank number: P[1]NCDV, AB119636; P[2]SA11, NC_011510; P[3]RRV, LC438917; P[4]DS1, CAD62680; P[5], GU565055; P[6]ST3, L33895; P[6]RV3, ADD31861; P[7]CRW-8, UniProtKB:P0C6Y8; P[8]Rotateq, GU565044; P[8]Wa, L34161; P[9]K8, D90260; P[10]69M, M60600; P[11]HRV, UniProtKB: B6RGK2; P[11]BRV, M92986; P[12]H2, D13397; P[13]A46, AY050274; P[14]HAL1166, L20875; P[15]LLR, JQ013506; P[16], KJ477163; P[17]993-83, D16352; P[18]L338, D13399; P[19]HumanMc345, D38054; P[20]EHP, U08424; P[21]HG18, AF237665; P[22]160-1, AF526374; P[23]A34, AY174094; P[24] TUCH, AY596189; P[25], KC140588; P[26], DQ061053; P[27], DQ242615; P28human, EU805773; P[29], AB454420; P[30], FJ169856; P[31], EU486962; P[32], FJ492835; P[33], AB513836; P[34], AB571047; P[35], EU486958; P[36], AB971763; P[37], JX204814; P[38], LC088110; P[39], LC088221; P[40], KT935478; P[41], KU128895; P[42], KX268757; P[43], KX268779; P[45], KU243630; P[46], KU243621; P[47], KX268790; P[48], KX814933; P[49], PKY905314; P[50], KY971955; P[51], MH285840.

VP4 can be cleaved into two subunits, VP5* and VP8* (Larralde et al., 1991). VP8*, located at the distal terminal of the spike, is responsible for the virus-ligand interaction while VP5* facilitates the host cell penetration through the conformation rearrangement and membrane fusion (Figure 1) (Settembre et al., 2011). VP8* has been identified to interact with specific glycans in a P genotype dependent manner (Huang et al., 2012). Previously, 35 P genotypes were classified into five genogroups based on the VP8* sequences (Figure 2A) (Liu et al., 2012). The widely prevalent RVA P genotypes in humans such as P[8], P[4], P[6], and a rare P[19] genotype were classified in P[II] genogroup. P[9], P[14], and P[25] were grouped in P[III]. P[11] identified mainly in infants made P[IV] genogroup. The rare genotypes, P[17], P[30], P[31], and P[35] constituted P[V]. The remaining 23 genotypes, including P[3], P[7], all belonged to P[I] genogroup. We also constructed the phylogenetic tree of the 50 P genotypes using the VP8* sequences (Figure 2B). It is noticed that there are more lineages besides the former five branches. It was proposed that six new genogroups should be included, such as P[46]/P[29], P[49], P[5], P[28]/P[50], P[45]/P[37], P[20]/P[16] (Figure 2B). Here, we delineated recent advances in the structural basis for glycan recognition of RV VP8*s.

## Crystal Structures of RV VP8*s

The structures of VP8*s of ten genotypes spanning P[I] to P[IV] genogroups have been determined, including VP8*s of human and animal RVs. NMR and X-ray crystallography studies revealed that VP8* possessed a typical galectin-like fold with a two twisted β-sheets, βH and βK, separated by a shallow cleft (Dormitzer et al., 2002b) (Figure 2A). VP8* structures of animal RV strains, a porcine P[6] RV z84, a porcine P[7] RV CRW-8, a rhesus P[3] RV RRV, canine P[3] rotavirus strain K9, and bovine P[11] RV B223 have been determined (Dormitzer et al., 2002b; Blanchard et al., 2007; Mishra et al., 2018; Sun et al., 2018a) (Hu et al., 2015). The VP8* structures of human P[4] DS-1, P[8] Wa/Rotateq, P[6] RV-3, P[19] MC345, P[14] HAL1166, P[11] N155, P[9] K8, P[25] CAU12-2 are clear (Figure 2A) (Monnier et al., 2006; Blanchard et al., 2007; Hu et al., 2012; Hu et al., 2015; Yu et al., 2015; Sun et al., 2016; Liu et al., 2017; Sun et al., 2018a; Hu et al., 2018; Li et al., 2021).

**FIGURE 3:**
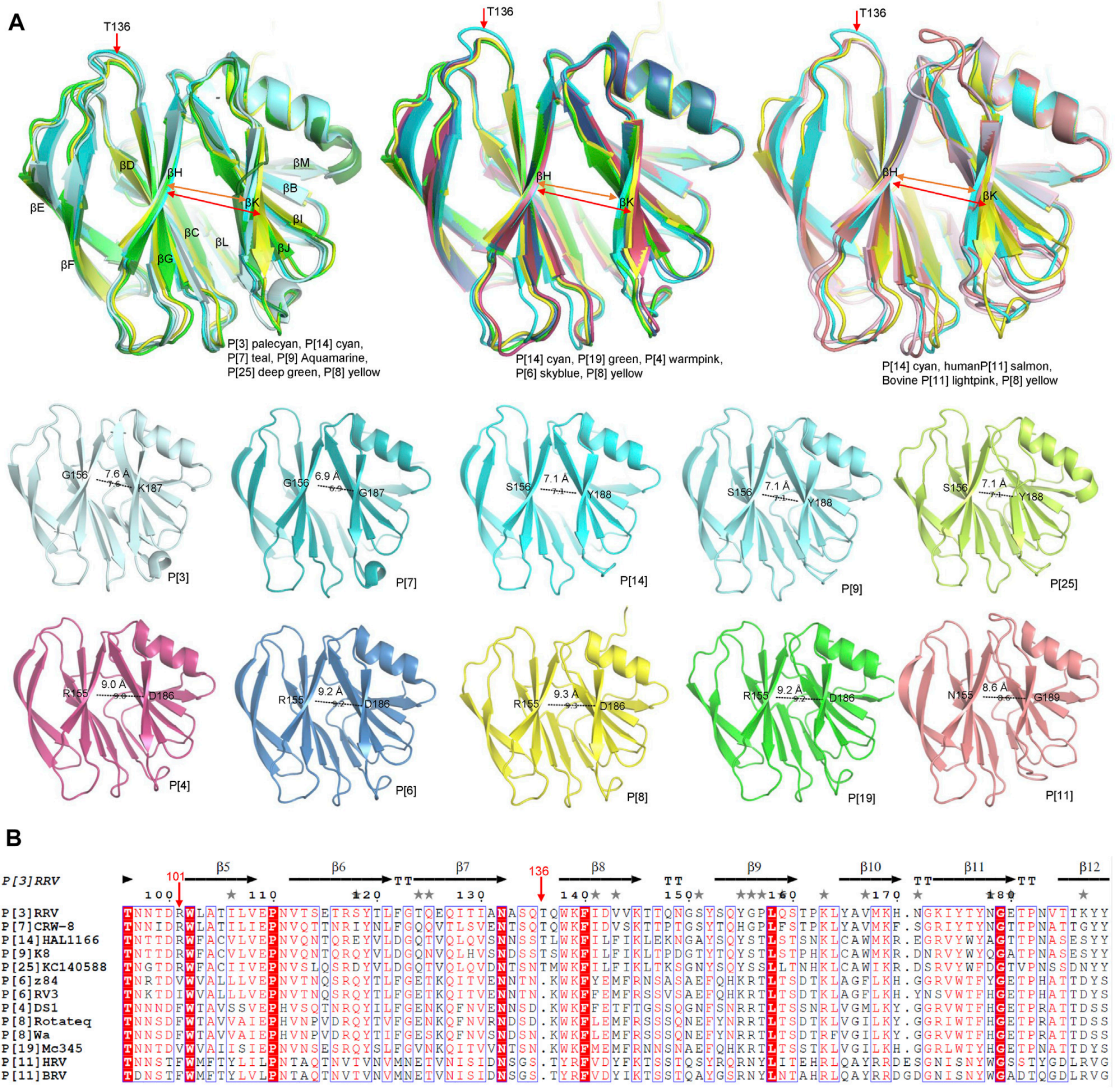
**(A)** Structural analysis of the VP8* structures. Superimposition of the VP8* structures to compare the width of the cleft between two β-sheets (βH and βK). The width of the cleft in VP8*s of different P genotype are measured. P[3], P[7], P[9], P[14], P[11], P[19], P[4], P[6], P[8] VP8*s are colored as described in Figure.2A and P[25] is colored deep green (PDB ID: 7C8P). **(B)** The structural based sequence alignment was constructed using Clustal Omega and displayed with the ESPript 3.0 (https://espript.ibcp.fr/ESPript/cgi-bin/ESPript.cgi). Residue 101 and 136 were pointed with red arrows.

P[8] and P[4] RVs are the most prevalent P genotypes in human infection (Lestari et al., 2020). P[6] RVs mainly circulate in humans and pigs (Nyaga et al., 2018). P[19], P[14], P[9], P[25] RVs are less common in humans and show evidence of cross-species transmission (Liu et al., 2012). P[11] RVs mainly infect neonates (Liu et al., 2013). The structural comparison showed that the galectin-like fold is conserved among these VP8* structures (Figure 2A). However, the widths of the cleft between the two β-sheets are different. Human P[8], P[4], P[6], P[19], P[11] VP8*s possessed a relatively wider cleft with the width of 9.3 Å (Ångstrom), 9.0 Å, 9.2 Å, 9.2 Å, 8.6 Å, respectively (Figure 3). Meanwhile, animal P[3] and P[7] VP8* possessed a narrower cleft with the width of 7.6 Å, 6.9 Å, respectively (Figure 3). Human P[14], P[9], P[25] RV VP8*s all possessed a relatively narrow cleft of 7.1 Å (Figure 3). As previously reported (Venkataram Prasad et al., 2014; Ramani et al., 2016), it was proposed that the deletion of residue 136 and the amino acid change at position 101 may in a certain part influence the width of the cleft based on the sequence alignment and structural analysis (Figure 3). P[3]/P[7]/P[14]/P[9]/P[25] VP8*s with narrow cleft had the R101 and 136T, while P[4]/P[6]/P[8]/P[19] with the F/V/I101 and 136 deletion possessed a wider cleft (Figure 3). Consistent with the phylogenic analysis of VP8* sequences, P[3] VP8* are structurally close to P[7] VP8* with the root mean square deviation (RMSD) value of 0.48 Å (Table 1), belonging to P[I] genogroup; P[8], P[4], P[6], and P[19] VP8*s classified in P[II] genogroup exhibited similar structural characteristics with the RMSD values ranging from 0.27 Å to 0.53 Å (Table 1); P[14], P[9], and P[25] VP8*s of P[III] genogroup presented alike conformation with RMSD value of 0.30 Å, 0.34 Å, and 0.39 Å (Table 1); P[11] VP8* grouped in P[IV] genogroup are distinct to all the other VP8* structures with the RMSD value above 0.75 Å (0.75–1.00 Å) (Table 1). These indicated that the VP8* structures possessed structural conformation in a genogroup dependent manner.

**TABLE 1:** The RMSDs (in Å) of the Cα atoms of VP8* monomer between different P genotypes.

R.M.S.D.	P[3]1KQR	P[7]3TAY	P[14]4DRV	P[9]5CB7	P[25]7C8P	P[4]5VX5	P[6]6OAI	P[8]6K2O	P[19]5YMT
P[3]1KQR									
P[7]3TAY	0.48								
P[14]4DRV	0.59	0.50							
P[9]5CB7	0.69	0.59	0.30						
P[25]7C8P	0.65	0.52	0.34	0.39					
P[4]5VX5	0.88	0.85	0.65	0.62	0.69				
P[6]6OAI	0.70	0.65	0.64	0.62	0.62	0.40			
P[8]6K2O	0.80	0.74	0.59	0.77	0.62	0.27	0.45		
P[19]5YMT	0.72	0.68	0.65	0.67	0.75	0.53	0.44	0.49	
P[11]4YG0	0.93	0.78	0.82	0.89	0.84	1.00	0.75	0.86	0.90

## Glycans Recognized by Rotaviruses

Some animal RVs were reported to recognize terminal sialic acids (SAs) (Fukudome et al., 1989; Isa et al., 1997). *N*-acetylneuraminic acid (Neu5Ac) and *N*-glycolylneuraminic acid (Neu5Gc) are the most common neuraminic acids in nature and widely expressed on the surface of most mammalian cells. Several RV strains of the P[I] genogroup, including Nebraska calf diarrhea virus (NCDV, cattle, P[1]), rhesus rotavirus (RRV, monkey, P[3]), SA11 (monkey, P[2]), porcine rotavirus strain (OSU, pig, P[7]) were sialidase sensitive and showed distinct preference for Neu5Ac or Neu5Gc (Rolsma et al., 1998; Dormitzer et al., 2002a; Isa et al., 2006). The infection of porcine P[7] RV CRW-8 could be efficiently inhibited by the ganglioside GM3Gc glycan (Blanchard et al., 2007). Canine K9 P[3] preferentially bound to Neu5Gc (Mishra et al., 2018). In addition, nuclear magnetic resonance (NMR) and cell infection studies showed that the ganglioside GM1, which lacks the terminal sialic acid but with branched sialic acid, could be a possible ligand for some human RVs, including human P[8] and P[6] (Haselhorst et al., 2009). VP8*s of human P[8] Wa and P[6] RV-3 were identified to bind to GM1 by Saturation transfer difference NMR (STD-NMR) (Fleming et al., 2014).

Later studies revealed that most animal RVs and human RVs are SA independent (Ciarlet and Estes, 1999). Recently, some human RV genotypes are found to recognize histo-blood group antigens (HBGAs) (Huang et al., 2012), indicating that HBGAs are important cell attachment factors for RVs. HBGAs are a group of carbohydrates (Yamamoto, 1994), distributing abundantly on mucosal epithelia. HBGAs also existed as free oligosaccharides in body fluids, such as saliva, milk, blood, and intestinal content. HBGAs are synthesized by sequential addition of monosaccharides to precursor disaccharides by different glycosyltransferase. The glycosyltransferases are encoded by three major gene families, secretor, Lewis, and ABO families encoding FUT2, FUT3, and A/B enzymes, respectively.

RV VP8*s recognized HBGAs in a genotype dependent manner. The P[8] and P[4] RVs that are widely prevalent in humans interacted with mucin cores, lewis b, and type I HBGA, including H type-1 antigen (H1), H1 precursor, lacto-N-tetraose (LNT), Lacto-N-fucopentaose I (LNFP1) (Huang et al., 2012; Rey et al., 2019; Sun et al., 2020). Human P[6] and P[19] bound to H1, whereas porcine P[6] and P[19] did not (Sun et al., 2018a; Li et al., 2018). P[14] was less common in human. Human P[14] VP8* was found to specifically recognize A type HBGA (Hu et al., 2012). Human P[9] and P[25], clustered in P[III] genogroup together with P[14] also bound to A type HBGA (Liu et al., 2012). Human P[11] RVs that mainly infected neonates interacted with type I and type II precursors, while bovine P[11] RVs only bound to type II precursor (Liu et al., 2013; Ramani et al., 2013; Hu et al., 2015). VP8* of human P[28] in P[I] genogroup was found to bind H1 HBGAs (Zhao et al., 2020). Bovine P[5] WC3 and its mono-reassortant G4P[5] recognized both sialic acid and the α-Gal HBGA (Alfajaro et al., 2019). Interestingly, the α-Gal epitope of the HBGA family was reported to be a ligand for bovine norovirus Newbury2 (Cho et al., 2018), indicating a common feature of the infection of certain bovine RVs and norovirus.

Mucins are large glycoproteins containing a protein core and a high number of O-linked oligosaccharides (Jensen et al., 2010). Mucin cores were reported to be recognized by several RV P genotypes. Human P[8] and P[4] RVs recognized the disaccharide core structure (GlcNAcβ1-6GalNAc) of mucin cores 2, 4, and 6 (Liu et al., 2016). Porcine P[6] (z84) VP8* interacted with mucin core 2, while human P[6] (5311142) VP8* did not bind to mucin core 2 (Sun et al., 2018a). Human and porcine P[19] bound to mucin core 2 (Sun et al., 2018a). A rare genotype P[10] VP8* also interacted with mucin core 2 (Pang et al., 2018). These results indicated that mucin cores especially mucin core 2 may play an important role in the RV infection and interspecies transmission.

The glycan binding specificity influenced the host tropism and prevalence of RVs. Animal RVs could recognize sialic acid and mucin core 2, whereas human RVs bound to HBGAs and mucin cores. The RVs that can infect both human and porcine such as P[6] and P[19] showed distinct glycan binding preference. Porcine P[6] and P[19] VP8*s recognized mucin core 2, while human P[6] and P[19] VP8*s interacted with the H1 HBGA, indicating an evolutionary path from animal to human. P[11] RV VP8* recognized type I and type II precursors that are developmentally regulated in neonates, consistent with the fact that P[11] RVs are mainly identified in neonates. P[8] and P[4] infections were mainly identified in secretors and lewis positive children (Nordgren et al., 2014), consistent with that P[8]/P[4] RVs could interact with H1 HBGA and lewis antigen.

## Structural Basis for the Interactions of VP8* and Glycans

**FIGURE 4:**
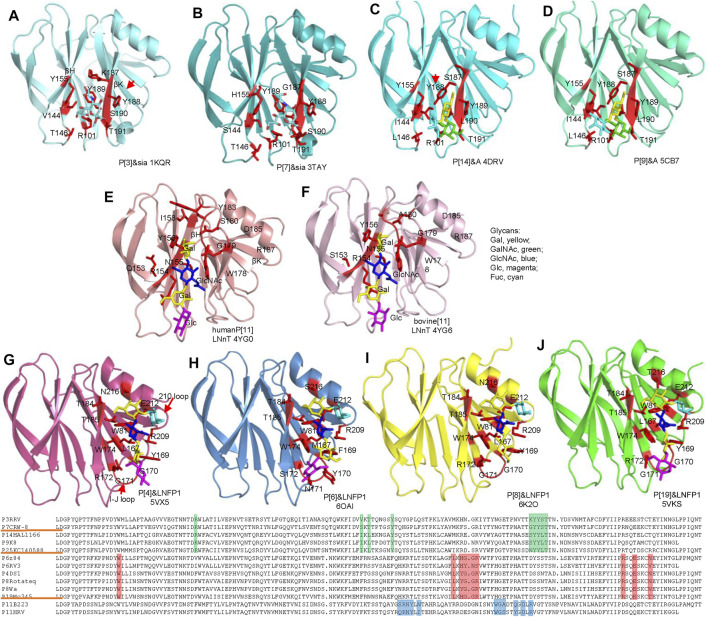
Structural analysis of different RV VP8*s in complex with glycans. VP8*s are shown as cartoon presentation. Glycans and amino acids participating the interactions are exhibited as sticks. RRV P[3] **(A)**, CRW-8 P[7] **(B)**, HAL1166 P[14] **(C)**, K8 P[9] **(D)**, human P[11] **(E)**, bovine P[11] **(F)**, P[4] Indian **(G)**, RV-3 P[6] **(H)**, Rotateq P[8] **(I)**, human P[19] **(J)**, VP8*s are colored as that in Figure.2A. The moieties of Gal, GalNAc, GlcNAc, Glc, Fuc are colored yellow, green, blue, magenta, cyan, respectively. Sequence alignment was done by DNAMAN. The residues involved in the glycan binding in P[3]/P[7]/P[14]/P[9], P[4]/P[6]/P[8]/P[19], P[11] VP8*s are colored green, red, blue, respectively.

The crystal structures of VP8*and VP8*/glycans are listed in table 2. Rhesus rotavirus (RRV) VP8* with sialic acid was first determined revealing a glycan binding site consisting of R101, Y155, 187-190 KYYS (Dormitzer et al., 2002b) (Figure 4A). The glycan binding site located at one corner of the cleft between two β-sheets (βH/βK) and appeared to be an open-shallow groove. Y188 and S190 form one rim of the groove; Y155 constitutes the other rim; R101, V144, K187 and Y189 side chains make the base part. R101 was proved to be vital for the sialic acid binding (Kraschnefski et al., 2009). CRW-8 VP8* interacted with Neu5Acα2Me using the same residues except H155 and G187 (Blanchard et al., 2007) and bound to ganglioside GM3 glycans by the same pattern (Yu et al., 2011). CRW-8 and RRV VP8* binding to the Neu5Gcα2Me were determined (Yu et al., 2012) (Figure 4B), illustrating that residue 157 of VP8* influenced the glycan preference. CRW-8 VP8* with S157 showed reduced binding affinity for Neu5Gc compared to that with P157.

**TABLE 2:** Summary of VP8* and VP8*-glycan structures. The PDB ID, glycan formula, and references are included. Gal, yellow; GlcNAc, blue; GalNAc, green; Glc, magenta; Fuc, cyan.

VP8* structure	PDB ID	Glycan formula	References
P[8] Wa	2DWR		Blanchard et al. (2007)
P[8] Rotateq	5JDB		Sun et al. (2016)
P[8] Rotateq and LNFP1	6K2O	Fucα1-2Galβ1-3GlcNAcβ1-3Galβ1-4Glc	Sun et al. (2020)
P[8] Rotateq and core2	6K2N	Galβ1-3(GlcNAcβ1-6)GalNAc	
P[8]_c_	6H9W		Rey et al. (2019)
P[8]_c_	6H9Z		
P[8] _c_ and LNB	6H9Y	Galβ1-3GlcNAc	
P[8] _c_ and H1	6HA0	Fucα1-2Galβ1-3GlcNAc	
P[4] DS-1	2AEN		Monnier et al. (2006)
P[4] Indian	5VX4		Hu et al. (2018)
P[4] Indian and LNFP1	5VX5	Fucα1-2Galβ1-3GlcNAcβ1-3Galβ1-4Glc	
Human P[6] RV3	5VX8		
P[6] RV3 and LNFP1	5VX9	Fucα1-2Galβ1-3GlcNAcβ1-3Galβ1-4Glc	
P[6] porcine z84	5YMU		Sun et al. (2018a)
P[6] BM11596	6NIW		Xu et al. (2020)
P[6] BM11596 and LNFP1	6OAI	Fucα1-2Galβ1-3GlcNAcβ1-3Galβ1-4Glc	
P[19] Mc345	5GJ6		Sun et al. (2016)
P[19] and Core2	5YMS	Galβ1-3(GlcNAcβ1-6)GalNAc	Sun et al. (2018b)
P[19] and LNT	5YMT	Galβ1-3GlcNAcβ1-3Galβ1-4Glc	
P[19] and core 2	5VKI	Galβ1-3(GlcNAcβ1-6)GalNAc	Liu et al. (2017)
P[19] and LNFP1	5VKS	Fucα1-2Galβ1-3GlcNAcβ1-3Galβ1-4Glc	
P[9] K8	5CAZ		Yu et al. (2015)
P[9] K8 and A	5CB7	GalNAcα1-3(Fucα1-2)Gal	
P[14] HAL1166	4DRR		Hu et al. (2012)
P[14] and A trisaccharide	4DRV	GalNAcα1-3(Fucα1-2)Gal	
P[14] and A tetrassacharide	4DS0	GalNAcα1-3(Fucα1-2)Galβ1-4GlcNA	
P[25] human	7C8P		Li et al. (2021)
P[7] porcine CRW-8	2I2S		Blanchard et al. (2007)
P[7] CRW-8 GM3	3SIT	Neu5Acα2-3Galβ1-4Glc	Yu et al. (2011)
P[7] CRW-8 and GM3-Gc	3SIS	Neu5Gcα2-3Galβ1-4Glc	
P[7] CRW-8_S157 and Neu5Gc	3TAY	Neu5Gc	Yu et al. (2012)
P[7] Porcine RV TFR-41	5CA6		Yu et al. (2015)
P[3] RRV VP8	1KRI		Dormitzer et al. (2002a)
P[3] RRV and MNA	1KQR	Neu5Ac	
P[3] RRV MNA 100K	2P3K	Neu5Ac	Kraschnefski et al. (2009)
P[3] RRV R101A	2P3J		
P[3] RRV 295K	2P3I		
P[3] RRV Neu5Gc	3TB0	Neu5Gc	Yu et al. (2012)
P[11] bovine B223	4YG3		Hu et al. (2015)
P[11] B223 and LNnT	4YG6	Galβ1-4GlcNAcβ1-3Galβ1-4Glc	
P[11] human N155	4YFW		
P[11] N155 and LNnT	4YG0	Galβ1-4GlcNAcβ1-3Galβ1-4Glc	
P[11] N155 and LNT	4YFZ	Galβ1-3GlcNAcβ1-3Galβ1-4Glc	
RVC human Bristol	5ZHG		Sun et al. (2018a)
RVC and A trisasscharide	5ZHO	GalNAcα1-3(Fucα1-2)Gal	

The structural basis of P[14] interacting with A type HBGA has been illustrated (Hu et al., 2012) (Figure 4C). The width of the cleft between the two β-sheets is narrower than the cleft in the human VP8*, similar to that in the VP8* of the animal strains (Figure 3). P[14] VP8* bound to the A type HBGA using the same glycan binding site as P[3] VP8*. However, the structural features of the glycan binding site of P[14] VP8* is that of P[3] VP8*. The amino acid residues involved the A type HBGA binding were R101, I144, L146, Y155, S187, Y188, Y189, and L190 (Figure 4C). The orientation of Y188 was different to Y188 in P[3] VP8* and would cause steric hindrance to the binding of sialic acid, indicating that the subtle changes of the VP8* could accommodate distinct glycans. The terminal GalNAc (green) and Gal (yellow) of the HBGA contributed to all the interactions, whereas the proximal moiety Fuc (cyan) project out from the surface and did not make any direct contacts (Hu et al., 2012). Human P[9] was identified to recognize A type HBGA using the same glycan binding site as that of P[14], consisting of R101, I144, L146, Y155, S187, Y188, Y189, L190, and T191 (Figure 4D). The amino acids possessed identical conformation as those in P[14] VP8*, providing further evidence that they belong to the same genogroup.

P[11] VP8*, clustered in P[IV] genogroup, possessed a quite different conformation comparing to other VP8*. Human P[11] VP8* interacted with type I and type II precursors using a distinct glycan binding site consisting of N153, R154, N155, Y156, I158, W178, G179, S180, Y183, D185, and R187 (Hu et al., 2015) (Figure 4E). The type I tetrasaccharide lacto-N-tetraose (LNT) and type II tetrasaccharide lacto-N-neotetraose (LNnT) binding site was expansive and spanned almost the entire length of the cleft between βH and βK (Figure 4E). Bovine P[11] only interacted with type II precursor and recognized LNnT with residues of S153, R154, N155, Y156, W178, G179, A180, D185, and R187 (Figure 4F).

**FIGURE 5:**
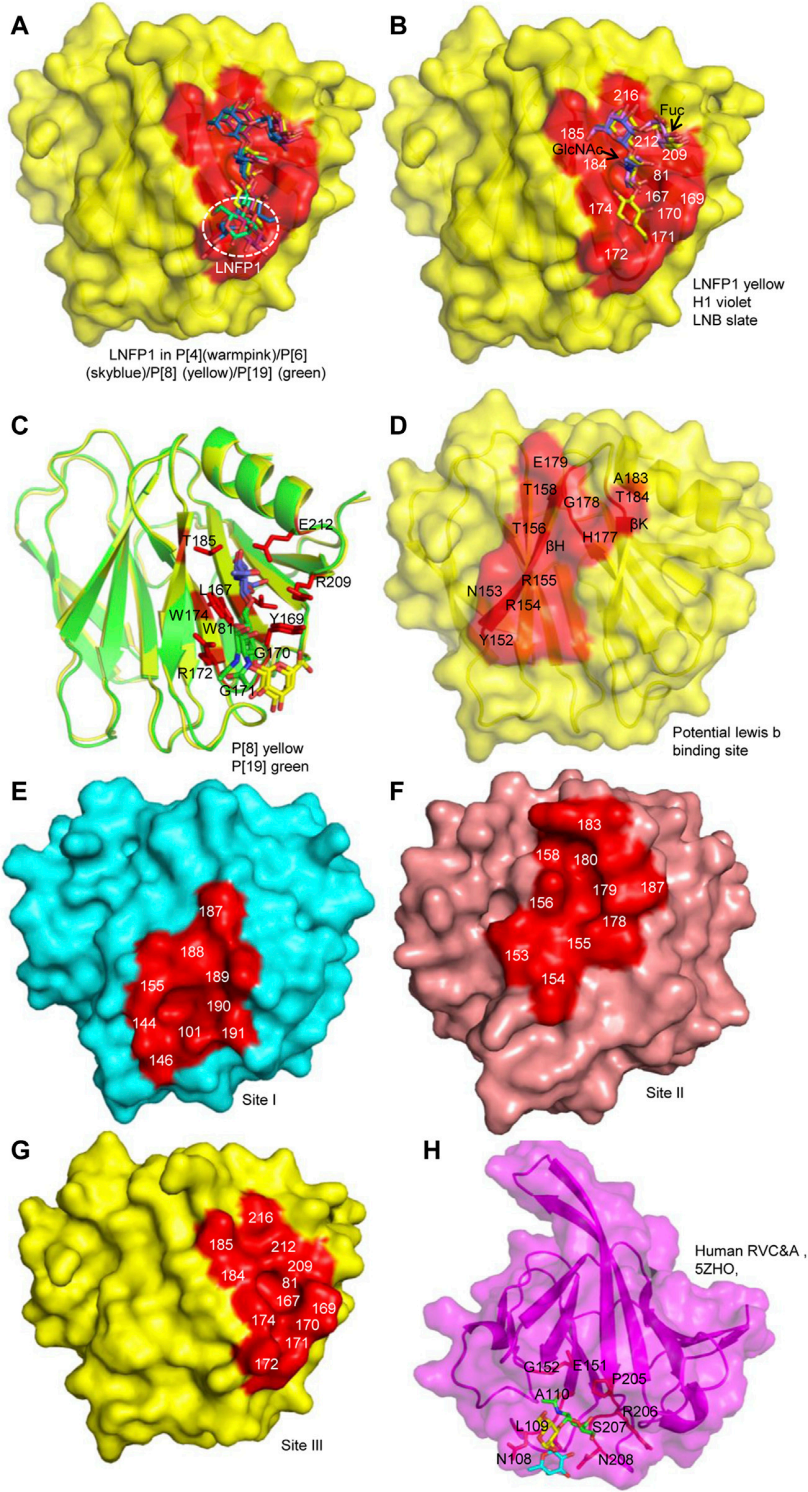
Comparison of the glycan binding sites. **(A)** The superimposition of LNFP1 that bound to P[4]/P[6]/P[8]/P[19] VP8*s. **(B)** Superimposition of type 1 HBGAs (H1, LNB, LNFP1) that located at the same glycan binding site of P[8] VP8*. **(C)** The interactions between P[8]/P[19] VP8* and mucin core 2. **(D)** The potential lewis b binding site in P[8] VP8* based on NMR. **(E–G)** Surface presentation of the three distinct glycan binding sites in RVA VP8*s. The residues constitute the glycan binding site were labeled. P[14] (cyan, 4DRV), human P[11] (salmon, 4YG0), P[8] (yellow, 6K2O) VP8*s are presented as models. **(H)** The presentation of the glycan binding site of human group C RV (RVC) VP8* (magenta, PDB ID: 5ZHO). Type A trisaccharide was shown as stick. The residues involved in the glycan binding site are labeled.

Human P[4]/P[6]/P[8]/P[19] VP8*s belonging to P[II] genogroup all interacted with type I HBGA (Figures 4G–J). Human P[4] and P[6] VP8*s with LNFP1 have been determined separately (Hu et al., 2018; Xu et al., 2020) (Figures 4G,H). The type I HBGAs located at a novel site consisting of the β-strand K, I-J loop, and 210 loop. Residues W81, L167, YGGR 169-172, W174, T184, T185, R209, E212, and N216 are involved in the glycan binding of human P[4] VP8* (Figure 4G). The LNFP1 binding site in human P[6] VP8* was at the same location but formed by different amino acids of W81, M167, FYNS 169-172, W174, T184, T185, R209, E212, and S216. The LNFP1 binding site in P[8] was identical to that in P[4] VP8* (Figures 4I,G), consistent with that they are close in the phylogenetic analysis. GlcNAc of LNFP1 contributed to the main interactions. Gal also participated in the interactions, whereas FucI was not involved in direct interactions (Figure 4I). The glycan binding site of P[19] VP8* is composed of W81, L167, YGGR 169-172, W174, T184, T185, R209, E212, and T216 (Figure 4J). Complex structures of P[19] VP8* with LNT and LNFP1 have been determined separately. The moieties of LNT, Gal1 (yellow), GlcNAc2 (blue), Gal3 (yellow), Glc4 (magenta) all participated in the interactions. LNFP1 located at the same place and almost overlap with the LNT. Fucose (cyan) of LNFP1 did not contribute to the binding. Superimposition of the complex structures of P[4]/P[6]/P[8]/P[19] VP8*s with LNFP1 revealed that LNFP1 overlapped at the same place in a similar conformation (Figure 5A). Only the orientation of the moiety of Glc at the reducing end displayed some variation (Figure 5A), revealing the exquisite of the interactions of different VP8*s and glycans.

The complex structures of P[8] VP8* with different type I HBGAs (H type-1 antigen, H1; H1 precursor lacto-N-biose, LNB; Lacto-N-fucopentaose I, LNFP1) were determined (Rey et al., 2019; Sun et al., 2020). LNB and H1 located at the same site of LNFP1 (Figure 5B). LNB and H1 interacted with VP8* using similar mechanism. L-fucose of H1 was projected out and did not make direct interactions with VP8*. However, the surface plasmon resonance (SPR) assay showed that P[8]_c_ VP8* bound more intensively to H1 (affinity constants K_D_ = 27.9 ± 0.7 μM) compared to LNB (K_D_ = 52.1 ± 4.3 μM) (Rey et al., 2019), implying that H1 L-fucose contributes to the glycan binding. LNFP1 overlapped exquisitely with the H1 and LNB moieties. P[8] VP8* interacted with different H1 glycans in a same site but with different binding affinity, indicating that the glycan forms may influence the RV attachment.

The interactions between P[8]/P[19] VP8* and mucin core 2 have been illustrated (Liu et al., 2017; Sun et al., 2018a; Sun et al., 2020). Mucin core 2 interacted with VP8*s at the same site as the type I HBGAs with slightly difference. GlcNAc (blue), GalNAc (green), and Gal (yellow) all participated in the interactions (Figure 5C) (Sun et al., 2020). P[8]/P[19] VP8* bound to mucin core 2 using the same pattern with residues of W81, L167, Y/HGGR 169-172, W174, T185, R209, and E212 (Figure 5C), revealing that RV VP8* can accommodate different glycans using the same residues.

P[8] and P[4] VP8*s were also reported to interact with lewis b (le^b^) (Huang et al., 2012; Ma et al., 2015). A recent paper elucidated the molecular mechanism for the recognition of P[8] VP8* to le^b^ based on nuclear magnetic resonance (NMR) spectroscopy-based titration experiments and NMR-derived high ambiguity driven docking (HADDOCK) method (Xu et al., 2020). Unlike the H1 binding site composed of an α-helix and a β-sheet (referred as βα binding site), P[8] and P[4] VP8*s were identified to bind le^b^ HBGA in another pocket consisting of the edge of two β-sheets (named ββ binding site) (Figure 5D). The potential lewis b binding site is proposed to be formed by residues of Y152, N153, R154, R155, T156, T158, H177, G178, E179, A183, and T184 (Figure 5D). Further investigations such as X-ray crystallization are needed to verify the glycan binding.

According to the crystal structures of VP8* and glycans, three glycan binding sites are identified in different RV VP8*s so far (Figures 5E–G). The first one is comprised of residues 101, 144, 146, 155, 187-191 locating in P[3]/P[7]/P[14]/P[9] (Figure 5E). The second one is that of P[11] RV VP8* (Figure 5F). The third one includes residues of 81, 167, 169-172, 174, 184, 185, 209, 212, 216 and is relatively conserved in P[II] genogroup RVs (Figure 5G). Based on the understanding of the importance of VP8* in RV-host interactions, the VP8*-based subunit vaccine has been explored. One VP8* vaccine has shown a good ability to induce neutralizing antibodies in immunized mice (Xue et al., 2015). VP8* was also presented on norovirus P particle to construct a P24-VP8* nanoparticle (Ramesh et al., 2019). A chimeric VP8* with T-cell epitope P2 exhibited better effect in inducing antibodies and protection than vaccines without P2 epitope. Polyvalent P2-VP8* vaccine candidates containing VP8*s of P[4], P[6] and P[8] are under trial (Groome et al., 2020). The VP8*-based vaccines probably be promising alternatives for future vaccines.

## Other Group Rotaviruses

Group/species C rotaviruses (RVCs) have been identified as important pathogens of acute gastroenteritis in children, family-based outbreaks, as well as animal infections (Joshi et al., 2017; Vlasova et al., 2017). Human RVC VP8* was found to recognize A type HBGAs (Sun et al., 2018b). The complex structure of human RVC VP8* and type A trisaccharide exhibited that human RVC VP8* possessed a completely different glycan binding site compared to RVA VP8*s (Figure 5H). Human RVC bound to type A trisaccharide (GalNAcα1-3(Fucα1-2)Gal) using a pocket consisting of N108, L209, A110, E151, G152, P205, R206, S207, and N208 (Sun et al., 2018b). Both GalNAc and Fuc of the type A HBGA participated in the interactions, while Gal had no direct contact with the RVC VP8*.

Human group B (RVB) and group H rotavirus (RVH) caused outbreaks in China in the 1980s and mainly infected adults. Infections of human RVB and RVH have constantly reported in some areas such as Southeast Asia (Chen et al., 1985; Yang et al., 2004; Jiang et al., 2008; Joshi et al., 2019). The receptor binding specificity of human RVB and RVH is unclear. Whether they recognize sialic acid as some animal RVAs, HBGAs as human RVAs or other glycans still need further investigation.

## Conclusion Remarks

Some animal RVs recognized sialic acid, such as P[3], P[7]. Some animal RVs were reported to bind sialic acid and αGal, such as bovine P[5] RVs (Alfajaro et al., 2019). The identification that some human RV VP8*s recognized HBGAs has provided new insights into the infection and transmission of RVs. So far, the interactions between VP8*s of human P[4], P[6], P[8], P[19], P[11], P[14], P[9], P[25] and HBGAs have been illustrated. In total, three distinct glycan binding sites were identified in different RVs based on crystallography. VP8* of the widely prevalent RV genotypes P[8], P[4], P[6], and a rare genotype P[19] VP8* possessed a conserved glycan binding pocket. Structural analysis revealed that the same glycan binding pocket could interact with different glycans exquisitely, such as that P[8] VP8* could accommodate H1, LNB, LNFP1, and mucin core 2. VP8*s of human RVA P[14], P[9], P[25] and human RVC all interacted with A-type HBGA, which may in a part restricted the prevalence of these RVs. The functions of these glycans, such as sialic acid, HBGAs, mucin cores in the RV infection or cross-species transmission still need more studies to clarify. Structural biology has significantly contributed to our understanding of the interaction between RV and glycans. However, the complexity and variety of glycan recognition of RV VP8*s indicated host-pathogen co-evolution with the structural and functional adaptation of RV to host glycan polymorphisms. More efforts exploring the structural basis for the VP8*-glycan interactions are necessary to fully understand the role of glycans in RV infection and transmission, which will facilitate the development of novel RV vaccines and anti-viral agents.
